# Type 1 diabetes human enteroid studies reveal major changes in the intestinal epithelial compartment

**DOI:** 10.1038/s41598-024-62282-x

**Published:** 2024-05-24

**Authors:** Vishwesh Bharadiya, Yan Rong, Zixin Zhang, Ruxian Lin, Anthony Lawrence Guerrerio, C. Ming Tse, Mark Donowitz, Varsha Singh

**Affiliations:** 1 Divisions of Gastroenterology and Hepatology, Department of Medicine, the Johns Hopkins University School of Medicine, Baltimore, MD 21205 USA; 2 Department of Pediatrics, The Johns Hopkins University School of Medicine, Baltimore, MD 21205 USA; 3 Department of Physiology, The Johns Hopkins University School of Medicine, Baltimore, MD 21205 USA

**Keywords:** Gastrointestinal models, Intestinal stem cells, Type 1 diabetes

## Abstract

Lack of understanding of the pathophysiology of gastrointestinal (GI) complications in type 1 diabetes (T1D), including altered intestinal transcriptomes and protein expression represents a major gap in the management of these patients. Human enteroids have emerged as a physiologically relevant model of the intestinal epithelium but establishing enteroids from individuals with long-standing T1D has proven difficult. We successfully established duodenal enteroids using endoscopic biopsies from pediatric T1D patients and compared them with aged-matched enteroids from healthy subjects (HS) using bulk RNA sequencing (RNA-seq), and functional analyses of ion transport processes. RNA-seq analysis showed significant differences in genes and pathways associated with cell differentiation and proliferation, cell fate commitment, and brush border membrane. Further validation of these results showed higher expression of enteroendocrine cells, and the proliferating cell marker *Ki-67*, significantly lower expression of NHE3, lower epithelial barrier integrity, and higher fluid secretion in response to cAMP and elevated calcium in T1D enteroids. Enteroids established from pediatric T1D duodenum identify characteristics of an abnormal intestinal epithelium and are distinct from HS. Our data supports the use of pediatric enteroids as an ex-vivo model to advance studies of GI complications and drug discovery in T1D patients.

## Introduction

Gastrointestinal complications are an important cause of morbidity in patients with type 1 diabetes (T1D)^1–3^. These include gastroparesis, abdominal distension, irritable bowel syndrome, fecal incontinence, diarrhea and constipation collectively called diabetic enteropathy (DE)^2,4^. These complications significantly reduce the quality of life in these patients, and importantly, are poorly understood mechanistically. Preclinical studies have identified significant changes in intestinal mucosal morphology in diabetic rodents^5,6^. However, our understanding of T1D-related GI pathophysiology in humans remains limited, in part due to the lack of robust *ex vivo* models that enable the interrogation of tissue structure, composition, stem cell function, and other changes in the epithelial compartment. Current treatments of T1D enteropathy are largely symptomatic and relatively ineffective. To develop targeted preventative therapy for T1D-related enteropathy, it will be important to establish models that appropriately represent the physiology of the human intestine while also maintaining disease pathophysiology.

The human intestinal epithelium is composed of polarized epithelial cells of different cell types that include enterocytes, enteroendocrine cells, tuft cells, goblet cells, Paneth cells, stem cells, and transit amplifying cells^7^. Intestinal stem cells maintain the expression of different cell types by controlled differentiation under normal physiologic conditions, while an imbalance in the expression of different epithelial cells occurs in response to multiple types of injury and physiological stresses^8^. Due to recent advances in in vitro stem cell culture that recapitulate many features of intestinal homeostasis, we can generate self-renewing three-dimensional ex vivo cultures, also called intestinal organoids or mini-guts^9,10^. These cultures contain the multiple cell types that make up the normal intestinal epithelial compartment. Intestinal organoids maintain 3D structure together with their self-renewing and differentiation capacities when embedded in Matrigel. Alternately, they can be fragmented or opened to form 2D epithelial monolayers (enteroids) when cultured on collagen-coated Transwell filters allowing for epithelial functional studies^11,12^. In some cases, including celiac disease and inflammatory bowel diseases, enteroids made from the affected intestine have been shown to maintain some aspects of the pathophysiology of the disorders^11,13,14^**.**

We have established duodenal enteroids from T1D patients and have compared them with age-matched HS and identified transcriptional and functional alterations in the intestinal epithelial compartment (IEC) in these patients. These alterations persist over multiple passages indicating persistent changes in intestinal stem cells. In this study, we demonstrated that T1D enteroids retain many IEC changes as reported in T1D patient samples of the intact intestine as well as in the intestine of the streptozotocin (STZ) rat model of T1D^6,15^. These changes include an increase in *Ki-67* expression indicating increased proliferation, an increase in enteroendocrine cells, a decrease in enterocyte differentiation, and a decrease in the Na^+^ absorptive protein sodium-hydrogen exchanger-3 (NHE3). In addition, IEC in T1D are highly susceptible to tight junctional disruption by Ca^2^^+^ deprivation and have higher fluid secretion in response to the known cAMP and Ca^2+^ stimulating agonists forskolin and ATP, respectively. This study suggests, for the first time, that enteroids developed from young T1D patients could be an appropriate preclinical model of T1D to understand abnormalities in the regulation of fluid and electrolyte imbalance that leads to altered bowel function and, importantly, understand mechanisms of multiple aspects of GI complications that occur in T1D patients.

## Results

### T1D enteroids have increased proliferation and an increased EE cell population

**Figure 1:**
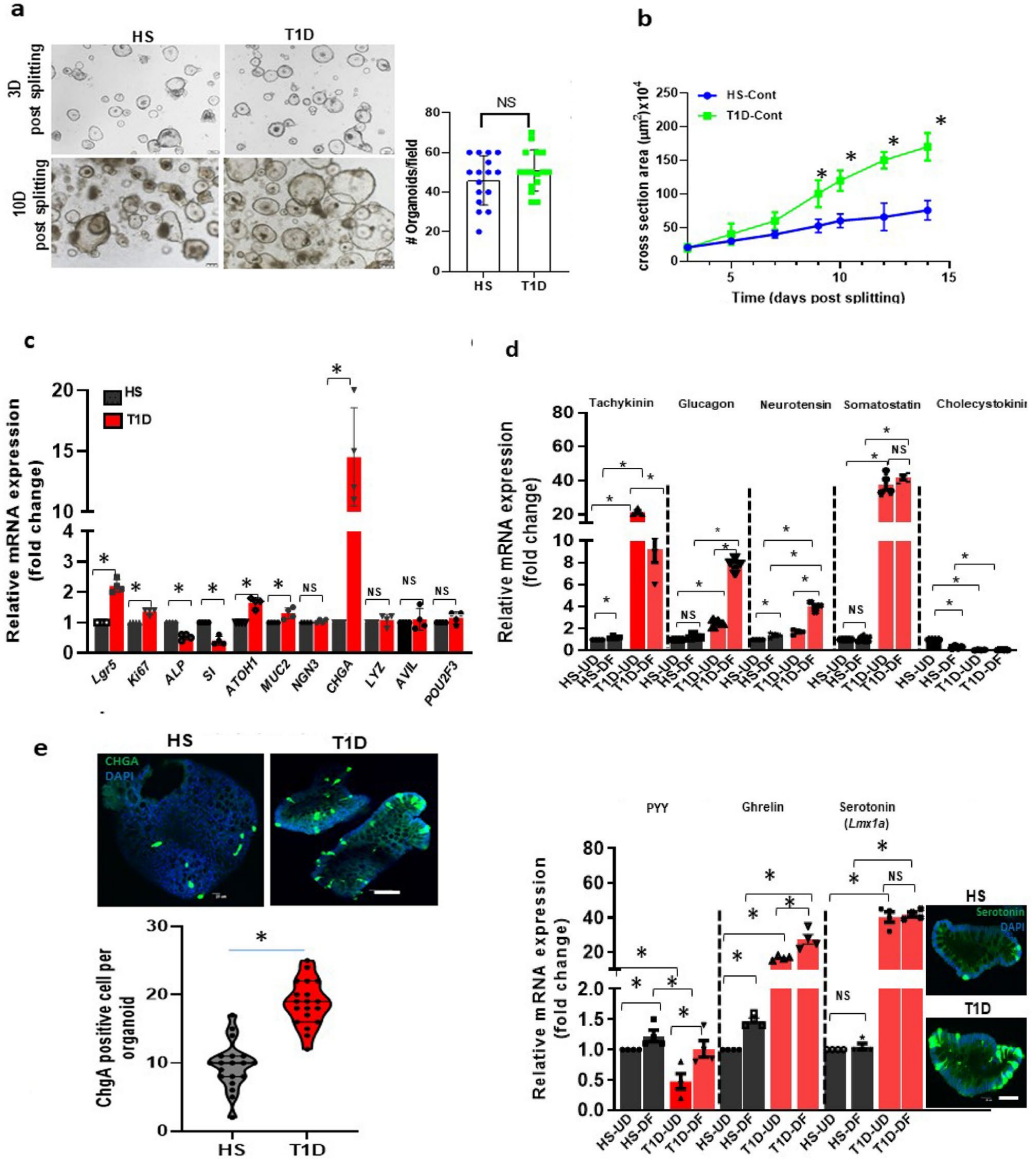
T1D duodenal enteroids have increased proliferation and an increased expression of the EE cell population. **(a)** A representative bright-field image of 3D duodenal enteroids from HS and T1D patients after 3 and 10 days post splitting and quantitation of the number of 3D enteroids formed at 3 days post splitting (right). (**b)** Quantitation of cross-sectional area showing larger-sized T1D duodenal enteroids at 10 days post-splitting from 30 fields from HS and T1D. Results in (**a**, **b)** are mean ± SEM, each data point represents the mean of 2–3 wells from each experiment from multiple donors. In (**b)**, results from three separate experiments including multiple enteroid lines are shown; ns = non significant; asterisks (*) represent *P* < 0 .05 vs HS calculated by Student’s unpaired t-test. **c.** qRT-PCR analysis in enteroids from HS and T1D patients (both UD) collected at 10 days post splitting showing differences in mRNA expression of genes between the groups. Results are shown as mean ± SEM of 3–4 independent analyses performed on each line. *P < 0.05 vs HS, NS, not significant. Analysis of differences was determined by Student’s unpaired t-test. (**d)** qRT-PCR analysis of mRNA isolated from HS and T1D enteroids in UD and 6-day DF stated showing differences in mRNA expression of hormones secreted from EE cell and changes with differentiation. *Lmx1a* mRNA was used as a marker for serotonin increase. Inset: A representative image from confocal microscopy from 6d-DF HS and T1D enteroid showing an increase in serotonin-positive EC cells (green) in T1D enteroids. Results are shown as mean ± SEM. *P < 0.05 vs control, NS, not significant. Analysis of differences was determined with a one-way ANOVA. N = 4–5 independent experiment was performed on enteroids from each donor. All measurements were considered in determining significance. **e.** Representative IF staining of 3D enteroid from 6d-DF HS and T1D enteroids stained with enteroendocrine cell marker, CHGA (green). Counterstain, Hoechst 33,342 (blue). The number of CHGA-positive cells was counted per organoid from 50 to 100 3D organoids from both T1D patients and HS and results are shown (right) as mean ± SEM; *P < 0.05 versus HS by Student’s unpaired t-test. Scale bar:50 μm. Data points in each represent means of 2–3 wells from each experiment from multiple donors.

The growth of T1D and HS enteroids was compared by counting the number of the 3D structures formed after each passage and the change in the size of the 3D structures over time. The growth of T1D enteroids based on the number of 3D spheroids formed per well after each passage was similar (Fig. 1a). However, T1D enteroids grew significantly larger than those from HS starting ~ 7 days after passaging (Fig. 1b). We also compared mRNA expression for markers of different cell types between HS and T1D. The qRT-PCR analysis of mRNA isolated from undifferentiated 3D enteroids collected on the 10^th^-day post-passaging showed a significant increase in mRNA expression of the enteroendocrine cell marker chromogranin A (*ChgA*) (Fig. 1c). To assess the state of enterocyte differentiation we compared the mRNA expression of epithelial stem cell marker Leucine-Rich Repeat Containing G Protein-Coupled Receptor 5 (*LGR5*) and the cell proliferation marker *Ki-67*, alkaline phosphatase (*ALP*) and sucrase-isomaltase (*SI*) (Fig. 1 c). Compared to HS, T1D enteroids had significantly higher expression of *Lgr5*, *Ki-67*, and lower expression of *ALP* and *SI* (Fig. 1c). In addition, T1D enteroids had activation of the secretory lineage pathway with increased mRNA expression of atonal bHLH transcription factor 1 (*ATOH-1*), the goblet cell (GC) marker-*MUC2* (~ 1.5 fold) and a very large increase in the EE cell marker-*ChgA* (~ 15 fold). Lysozyme (*LYZ*) was used as a marker for Paneth cells. Advillin (*AVIL*), and POU class 2 homeobox 3 (*POU2F3*) as markers for tuft cells were not significantly different between HS and T1D enteroids (Fig. 1c)^16^.

Since T1D enteroids had increased EE cells, we began the characterization of EE cell products to determine if specific EE cell subtypes were altered in T1D enteroids. Quantitative-RT-PCR was performed on undifferentiated (UD) and 6d-differentiated (6d-DF) enteroids. All EE cell products analyzed were significantly higher in T1D enteroids, except for *PYY* and *CCK*, which were significantly decreased (Fig. 1d). These differences occurred in both UD and DF enteroids (Fig. 1d). An increase in ChgA-positive EE cells was further confirmed using immunofluorescence (IF) analysis on 6d-DF 2D monolayers derived from T1D enteroids (Fig. 1e). Surprisingly, T1D enteroids did not have an increase in the mRNA expression in the EE cell progenitor marker Neurogenin 3 (*Ngn3*) (Fig. 1c). Enteroids from both T1D patients showed similar results. These results indicate that there are alterations in the IEC compartment in individuals with T1D and changes in the EE cell population are probably not due to *Ngn3-*related transcriptional changes.

### T1D enteroids have differences in the IEC transcriptomes

**Figure 2:**
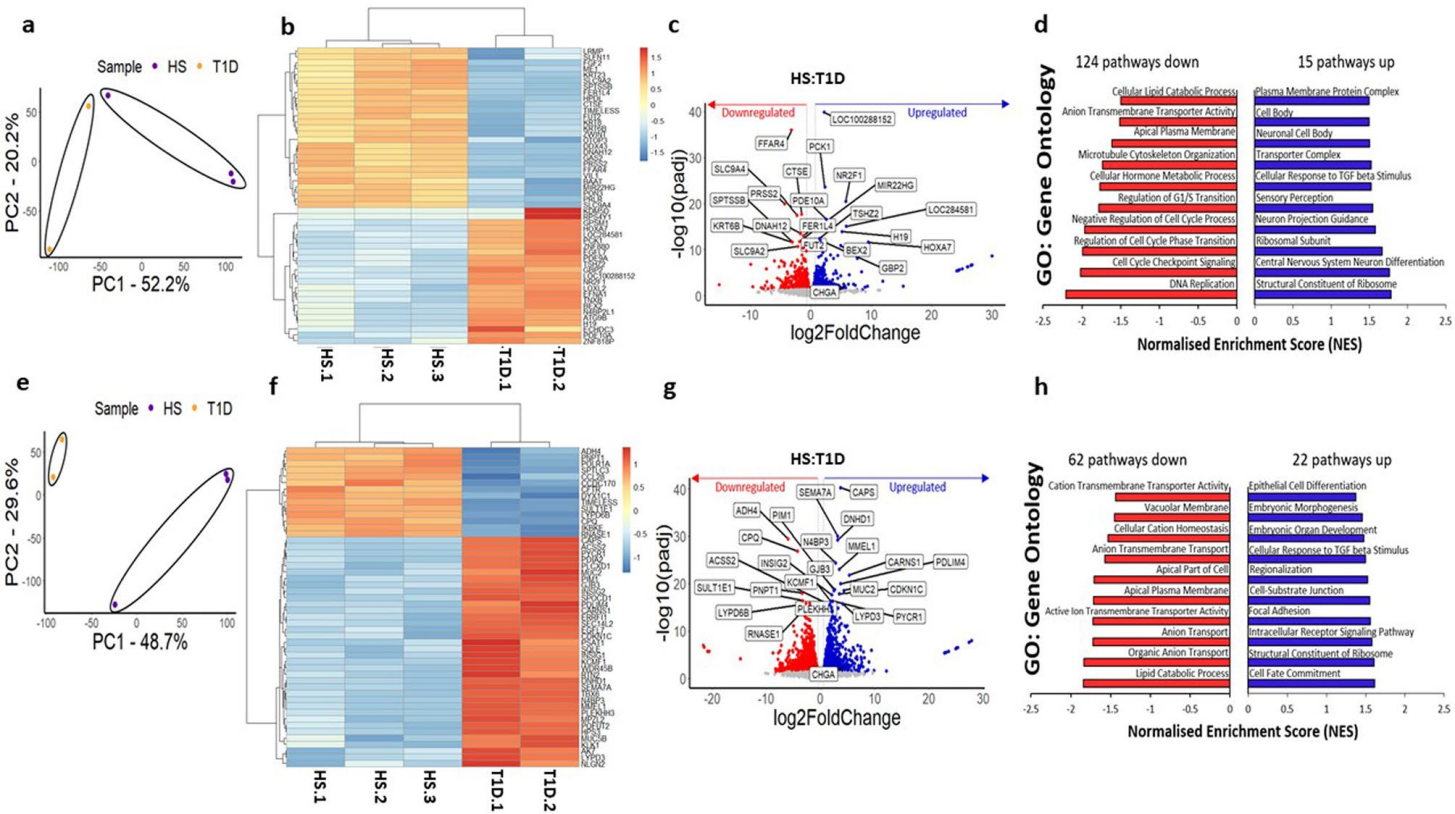
Transcriptional signatures of T1D enteroids are distinct from HS enteroids. Principal component analysis of: (**a)** UD, T1D over HS enteroids, and **(e)** 6d-DF, HS, and T1D enteroids, with the ellipses indicating clustering of the 2 groups. Heatmap of top 50 genes differentially expressed in **(b)** UD, T1D and HS enteroids, and **(f)** 6d-DF T1D and 6d-DF HS (fold change exceeding 1.5, p adj. < 0.5). Volcano plots of differential gene expression analysis results in UD (**c**) and 6d-DF (**g**) enteroids. Each gene is represented by a dot; the blue dots represent significantly up-regulated genes in T1D enteroids over HS, and the red dots represent significantly down-regulated genes in T1D enteroids over HS. The top 50 genes by statistical significance are annotated. Gene set enrichment analysis (GSEA) results using the Gene Ontology (GO) Biological Processes compendium showing significantly up- and down-regulated pathways in **(d)** UD and **(h)** 6d-DF T1D over HS enteroids.

To compare the transcriptional profiles of HS and T1D enteroids, bulk RNA sequencing (RNA-Seq) was performed on UD and 6-day differentiated duodenal enteroids grown as 3D cultures. Principal components analysis (PCA) was performed with normalized transcript per million (TPM) counts from the RNA-seq data, separately analyzing the UD and DF clusters. PCA showed a distinct clustering of the HS and T1D enteroids (Fig. 2a, e). Differentially expressed genes (DESeq2) between the HS and T1D clusters in both UD and DF samples were identified. In UD samples, we identified a total of 1779 differentially expressed genes (DEGs, fold change > 1.5, p-adj) in the T1D group compared with the HS group, including 778 up-regulated genes and 1001 genes that were down-regulated. In DF samples, there were 3384 DEG in the T1D group compared with the HS, including 1701 up-regulated genes and 1683 down-regulated genes. Heatmaps were plotted for UD and DF samples using the top 50 differentially expressed genes in each dataset (according to p-adj), with the gene names as row name labels shown using normalized counts from the DESeq2 analysis. Heat maps in both UD and DF samples showed separate clustering of HS from T1D samples in each suggesting disease-specific global transcriptional changes in T1D enteroids (Fig. 2b, f). A volcano plot representing all genes was constructed, with the DEGs represented with blue (upregulated) and red (downregulated), respectively. All significantly differentially expressed genes (p-adj < 10^–10^ for the undifferentiated samples, and p-adj < 10^–15^ for the differentiated samples) were labeled using gene names (Fig. 2c, g). Among the most significantly upregulated genes in the T1D enteroids compared with HS were genes related to the control of cell cycle progression: *DDX3Y* (DEAD/H (Asp-Glu-Ala-Asp/His) Box Polypeptide, Y), translation initiation factor: *EIF1AY* (Eukaryotic Translation Initiation Factor 1A Y-Linked), histone modification: *UTY* (ubiquitously transcribed tetratricopeptide repeat containing, Y-linked), *KDM5D* (lysine demethylase 5D), GTPase activator: *RGPD1* (RANBP2 Like And GRIP Domain Containing 1), and cAMP-dependent serine/threonine protein kinase activator: *PRKY* (protein kinase Y linked). The most significantly downregulated genes in T1D enteroids included genes related to: intracellular transport: *ATP6V0D2* (H + -ATPase, also known as vacuolar ATPase, V-ATPase), cell–cell communication: *GLDN* (Gliomedin: mediates interaction between Schwann cell microvilli and axons), *CXCL5* (C-X-C motif chemokine 5, also known as epithelial-derived neutrophil-activating peptide 78), immunosuppression: *IL19* (interleukin-19), and maintenance of intracellular pH: MCTs (Monocarboxylate transporters: *SLC16A6*, and *A14*). Importantly, most of the genes are common between UD and DF T1D enteroids. Gene set enrichment analysis (GSEA) using the gene ontology biological processes compendium (GOBP) on all expressed genes showed 124 (UD) and 62 (DF) downregulated pathways and 15 (UD) and 22 (DF) upregulated pathways in T1D enteroids. We performed gene set enrichment analysis to identify signaling pathways altered in T1D genes. The significant enrichment results (FDR < 0.05) are shown with normalized enrichment scores (NES) (Fig. 2 d, h). Some of the most physiologically important downregulated pathways of UD T1D enteroids were regulation of the G1/S transition, anion transport, and negative regulation of the cell cycle process, while in DF T1D, genes characterized as being present in the apical part of the cell, active ion transmembrane activity and vacuolar membrane pathways were downregulated in DF T1D. Together, these data demonstrate differences in the transcriptome of the epithelial compartment between enteroids from T1D and HS.

### T1D enteroids have changes in the expression of apical membrane proteins involved in nutrient absorption and anion secretion

**Figure 3:**
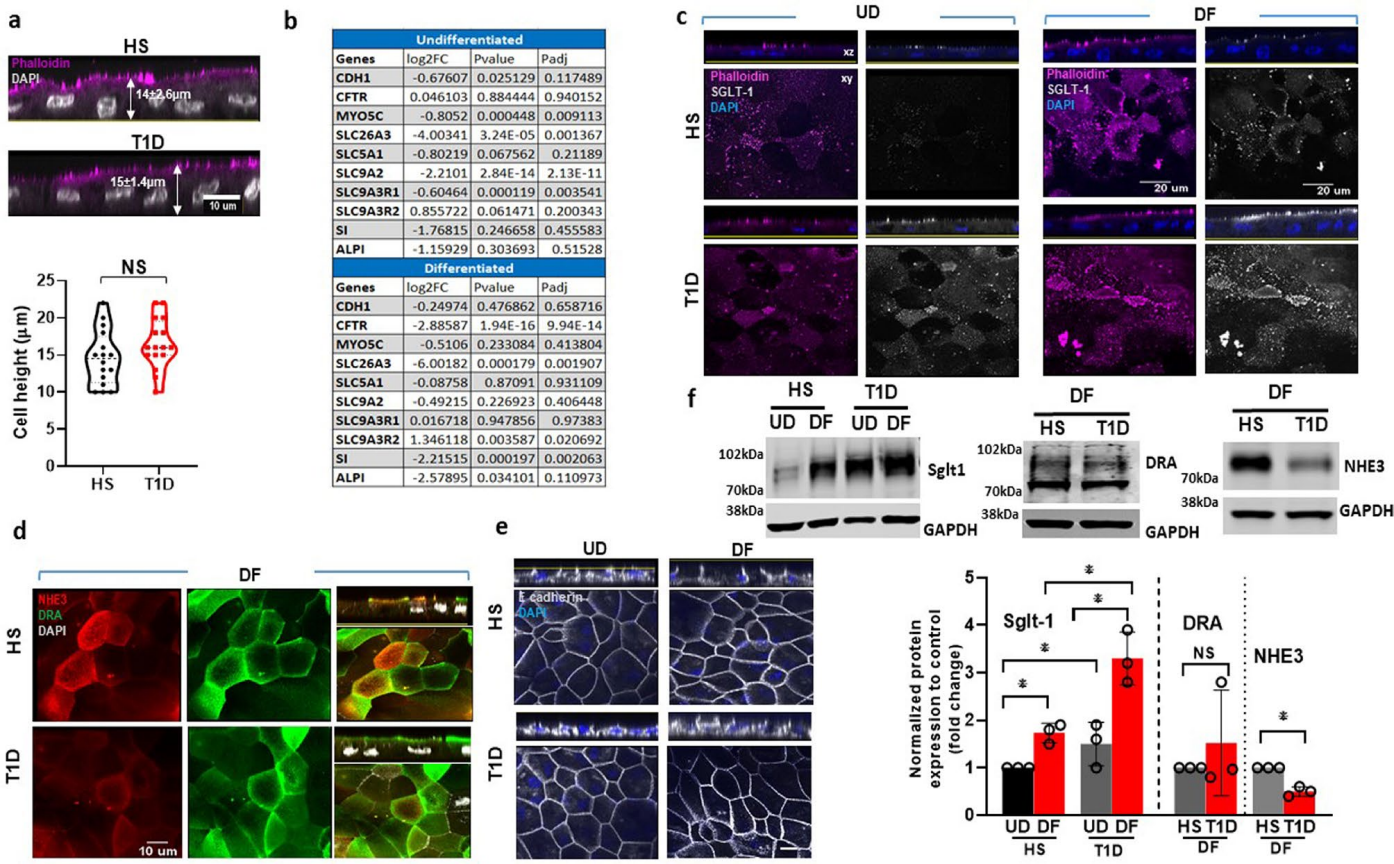
T1D enteroids have altered expression of apical membrane proteins involved in nutrient absorption and anion secretion. **(a)** Quantitation of cell heights measured in phalloidin and DAPI-stained 6d-DF enteroid monolayer from T1D and HS; NS = non significant calculated by Student’s unpaired t-tests. Scale bar, 10 μm. **(b)** RNA Sequencing data on several ion transport proteins/regulators expressed in T1D compared to HS enteroids. Representative immunofluorescence confocal images (XY and XZ) of monolayers immunostained for **(c)** SGLT1 (white), phalloidin (magenta), and nucleus (blue); **(d)** NHE3 (red), DRA (green), and nucleus (white). Scale bar, 20 μm; **(e)** E-cadherin (white), nucleus (blue). Images are representative of three independent experiments. Scale bar, 10 μm. **(f)** SGLT1, DRA, and NHE3, expression in total lysates prepared from UD (left) and 6d-DF (right) enteroids. A representative immunoblot (above) and densitometry (below) from three independent experiments is shown. Results are normalized to GAPDH used as a loading control and expressed as mean ± SEM of fold change; asterisks (*) represent *P* < 0.05 Student’s unpaired *t*-test.

Pathway analysis showed differences in the apical plasma membrane and apical part of the cell between DF T1D and HS enteroids. We first evaluated differences in the cellular morphology of enteroid lines through the measurement of single-cell heights from actin-stained 6-day DF monolayers on Transwell inserts. The cell height of DF T1D enteroids was not significantly different from HS (Fig. 3a). There were donor-specific differences in cell height within both groups but this interpretation was based on statistical analysis of data from multiple monolayers. To further compare the expression of apical membrane transport proteins, we analyzed the RNA-seq dataset for important solute and ion transport proteins and E-cadherin. Shown in Fig. 3b, UD T1D enteroids had reduced mRNA expression of myosin 5C, Down-Regulated in Adenoma (*DRA or Slc26a3*), *NHE2*, and sodium-hydrogen exchanger regulatory factor-1 (*NHERF1*) but no significant change in *E-cadherin*, Cystic fibrosis transmembrane conductance regulator (*CFTR*), sucrase-isomaltase (*SI*) or alkaline phosphatase-1 (*ALP1*). In DF enteroids, T1D had lower expression of *CFTR*, *DRA*, *SI* and increased expression of *NHERF2* but no significant change in expression of E-cadherin, myosin 5C, sodium-glucose cotransporter-1 (*SGLT-1*), *NHE2*, *ALP1* or *NHERF1* (Fig. 3b). We further determined changes in ion transporter protein expression via IF and western blotting performed on UD and 6-day differentiated enteroids monolayers on Transwell filters. Unlike the mRNA expression, the protein expression of SGLT-1 was increased in UD and 6d-DF T1D enteroids compared to HS (Fig. 3c, f). Protein expression of NHE3 was decreased in 6d-DF T1D enteroids compared to HS enteroids, while expression of E-cadherin and DRA were not significantly affected (Fig. 3d-f). Overall, these results indicate changes in ion transport protein expression in IEC in T1D, while changes in protein expression of SGLT-1 and DRA did not correlate with changes in their mRNA expression.

### T1D enteroid have normal TEER under basal conditions but have lower barrier integrity than HS

**Figure 4:**
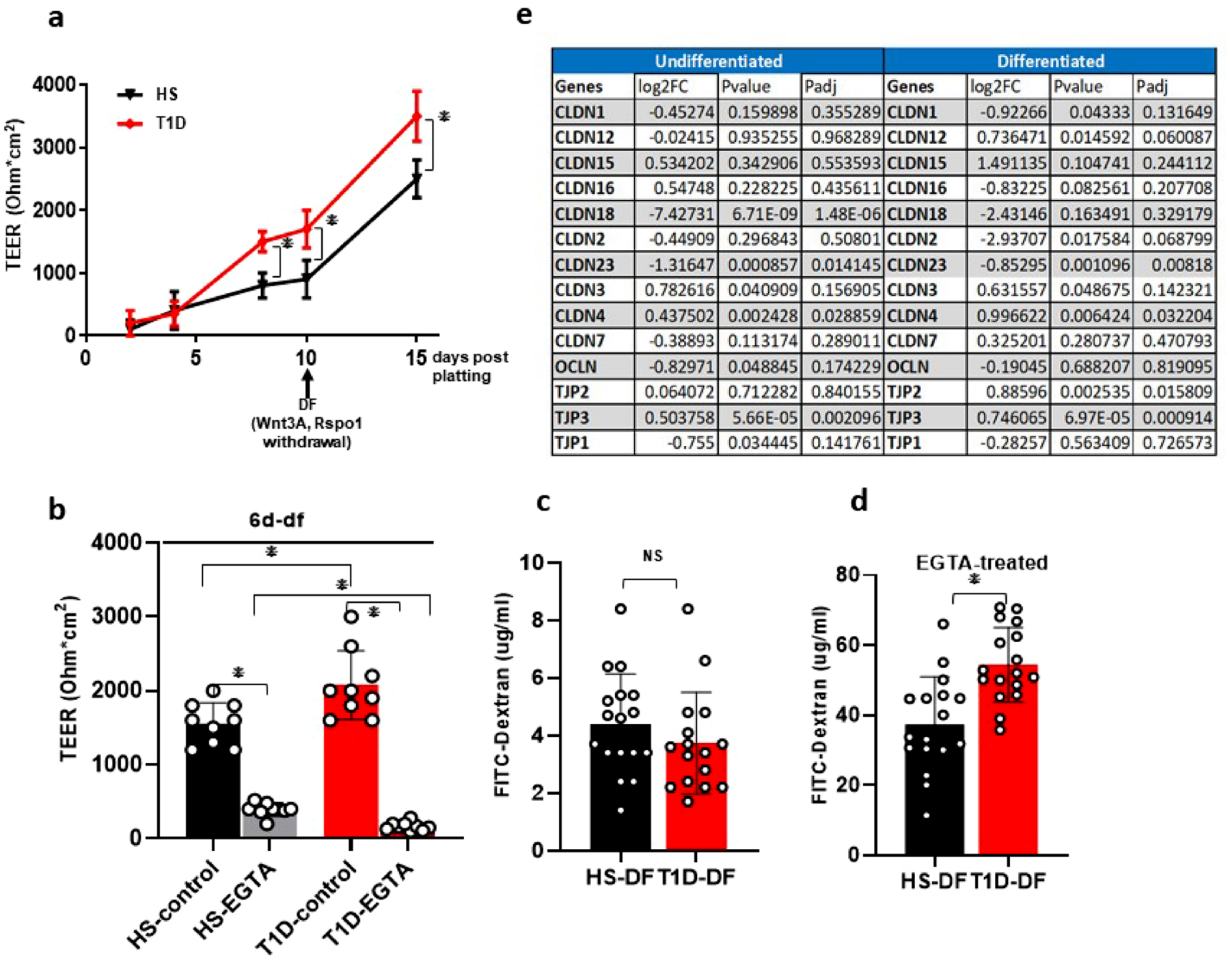
T1D enteroids have higher epithelial permeability than HS enteroids. **(a) **TEER values of enteroid monolayer at different days of confluency and increase with DF. Results are mean ± SEM; asterisks (*) represent P < 0.05 Student’s unpaired t-test. **(b)** TEER values of 6d-DF enteroid monolayers at baseline and after EGTA treatment. Results are mean ± SEM; * represents P < 0.05 Student’s unpaired t-test. **(c)** Flux of 4 kDa FITC-Dextran at baseline and **(d)** after EGTA treatment. Data represent mean ± SEM from four independent experiments, with each experiment including the two T1D and three HS lines. The P-values were calculated by Student’s unpaired t-test, and the asterisks (*) represent P < 0.05. **(e)** RNA Sequencing data showing differences in tight junction protein expression between T1D compared to HS enteroids.

Damaged intestinal barrier function is known in T1D^17^. To understand if T1D enteroids maintain barrier defects ex vivo*,* we assessed the barrier integrity of monolayers on Transwell inserts by measuring transepithelial electrical resistance (TEER). First, we evaluated the progress of monolayer formation by monitoring the steady increase in TEER^11,12^. Under basal conditions, starting at day-7 post-plating the TEER of UD T1D monolayers was significantly higher than the HS monolayer (Fig. 4a). Withdrawal of growth factors (Wnt3A and Rspon-1) caused an increase in TEER of both HS and T1D enteroids with T1D enteroids remaining significantly higher (Fig. 4a, b). We next evaluated functional differences in permeability by measuring 4 kDa fluorescein isothiocyanate (FITC)-dextran translocation across the enteroid monolayers. Cells were treated (2 h) with 5 mM ethylene glycol-bis(beta-aminoethyl ether)-N,N,N’,N’-tetra acetic acid (EGTA), a specific calcium chelator shown to have dramatic effects on paracellular permeability and TEER^18^. Treatment with EGTA caused a significant drop in TEER of both T1D and HS enteroids (Fig. 4b). However, T1D enteroids were more sensitive to barrier disruption with EGTA treatment compared to the HS (p < 0.05) enteroids (Fig. 4b). Under basal conditions, a similar amount of FITC-dextran translocated across the T1D (3.9 µg/ml) and HS monolayers (4.6 µg/mL) (p = NS) (Fig. 4c). In contrast, after EGTA treatment, significantly higher FITC-dextran translocation in the T1D monolayers (54.5 µg/mL) was detected compared to adult HS monolayers (37.9 µg/mL, p < 0.05) (Fig. 4d). To further characterize differences in barrier function, we analyzed differences in tight junction components in the bulk RNA-Seq dataset. In differentiated enteroids with more mature junctional complex, there were significant differences in the expression of tight junction proteins: T1D enteroids had significantly increased mRNA for claudin 4 (*CLDN4*), *ZO2*, and *3* but no change in *ZO1*; in addition, there was a significant decrease in expression of claudin 23 (*CLDN23*) (Fig. 4e). These data suggest alterations in tight junction protein expression and function in T1D enteroids.

### T1D enteroid have enhanced fluid secretion in response to forskolin and ATP

**Figure 5:**
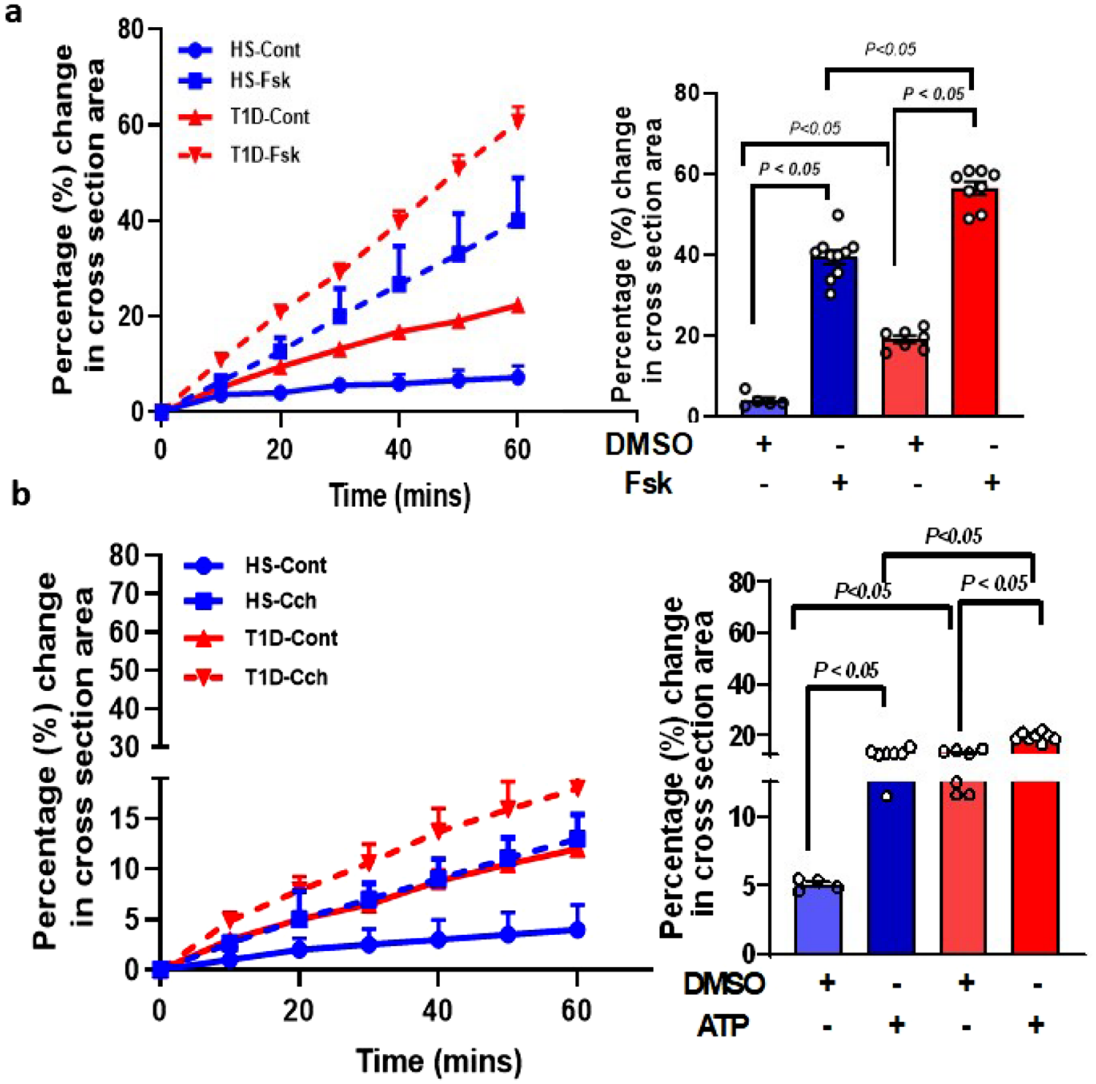
T1D enteroids have higher epithelial permeability than HS enteroids. Quantification of swelling response in UD enteroids from T1D and HS treated with: (**a)** forskolin (5 µM) and (**b)** ATP (Ca^2+^) (5 µM). Results are expressed as the percentage change in surface area relative to t = 0 (percentage change in cross-section area) measured at 10-min time intervals for 1 h (means ± SEM), and the bar graph depicts fluid secretion plots after subtracting basal (DMSO) secretion from the respective control (t = 60 min, means ± SEM, data points represent an average of 3–5 swelling assays each containing 50–100 enteroids from individual donors). Analysis of differences was determined with a one-way ANOVA and Bonferroni post hoc test.

To determine if there were differences in fluid secretion between HS and T1D enteroids, an in vitro functional assay based on Forskolin Induced-Swelling (FIS) assay on 3D UD enteroid was used; this method has been previously validated by us and others for studying intestinal fluid secretion^19,20^. As shown in Fig. 5a, b, the relative area increase is expressed per 10-min time interval, and measurements were generated for each condition (T = 0 to 60 min, baseline threshold set at 100%). DMSO-treated (control) HS enteroids showed no significant change in the surface area of enteroids while T1D enteroids showed significantly higher basal swelling compared to HS at 60 min (HS: 3.0 ± 1.3%, vs T1D: 19.0 ± 0.9%; p ≤ 0.05 vs HS) (Fig. 5a). Treatment of 5 µM Fsk caused a significant increase in the surface area of enteroids from both HS and T1D. The FIS response calculated after subtracting basal response was significantly higher in T1D enteroids compared to HS after 60 min of secretion (HS: 39.5 ± 1.6%, vs T1D: 56 ± 1.6%; p ≤ 0.05 vs HS; N = 3–5 assays on enteroids from each donor) (Fig. 5a). We also compared the fluid secretion in response to ATP (5 µM) which acts by elevating intracellular Ca^2+^^21^. DMSO-treated HS enteroids showed no significant swelling response while T1D enteroids showed a significantly higher basal swelling (HS: 5.0 ± 0.3%, vs T1D: 12.9 ± 0.4%; p ≤ 0.05). Compared to DMSO control, ATP caused a significant increase in the area of enteroids in both HS and T1D (HS: 13.1 ± 0.4%, p ≤ 0.05 vs DMSO at 60 min after agonist addition; and T1D:19.4 ± 0.5% p ≤ 0.05 vs DMSO), calculated after subtracting basal swelling response due to DMSO (Fig. 5b). Similar to the Fsk response, T1D enteroids had a significantly higher response to ATP compared to HS. These results showed that T1D enteroids had significantly higher fluid secretion in response to both cAMP and elevated Ca^2+^ compared to HS.

## Discussion

While no detailed transcriptomic or proteomic data have been reported for the small intestinal or colonic IEC of patients with T1D, there is a need to understand how changes in intestinal epithelial cell compartments contribute to the pathogenesis of T1D diarrhea, which remains an important cause of morbidity in these patients. A lack of biologically relevant model systems has limited the ability to perform mechanistic studies on the GI complications of T1D. Previous attempts to establish enteroid as a biologically relevant model using intestinal epithelial stem cells (IESC)-derived ex vivo cultures of IEC from T1D patients demonstrated that in patients with T1D who also had end-stage renal disease colonic stem cells were reduced in numbers and their self-renewal potential was decreased. This was attributed to increased levels of circulating insulin growth factor binding protein (IGFBP3)^22^. Shown here, we have successfully established duodenal enteroids from younger T1D patients who did not have end-stage renal disease and demonstrated that they could be passaged at least 30 times while retaining a consistent pathology as indicated by changes in mRNA expression retained in passage 30 (Supplementary Fig. 1a). These enteroids differ from those from normal HS by exhibiting increased expression of the proliferation marker *Ki-67* and *LGR5* indicating an increased proliferative compartment. In addition, T1D enteroids had altered enterocyte differentiation indicated by the reduced NHE3 and *SI*. In addition, there was a shift in the differentiating secretory cell composition with increased expression of the EE cell marker *ChgA* and goblet cell marker *MUC2* while there did not appear to be changes in Paneth cell or tuft cell expression (Fig. 1c). The alteration in the IEC cell compartment in enteroids and the consistency of these findings among many passages indicate a likely possibility that epigenetic changes in ISC in T1D contribute to an imbalance in the secretory and absorptive enterocyte populations.

Relevant to the abnormal bowel habits, particularly diarrhea that occurs in T1D patients, was our observation of an increased enteroendocrine population in the T1D enteroids. The total number of ChagA-expressing cells was markedly increased in T1D duodenal enteroids as were the mRNAs for tachykinin (substance P), glucagon, neurotensin, ghrelin, and somatostatin while the only EE products that were reduced were PYY and the low level of CCK that was expressed. In addition, LIM homeobox transcription factor 1 alpha (*Lmx1a*) known to control serotonin biosynthesis in the intestine together with serotonin-positive EE cells was increased in T1D enteroids^23^. Most of these changes occurred in both UD and DF enteroids. Of these hormonal substances, elevated serotonin, substance P, and neurotensin are known to cause changes in intestinal transport contributing to the pathophysiology of diarrhea, while somatostatin is suggested to inhibit multiple forms of secretory diarrhea^24,25^. EE cell types are usually classified based on their hormone production: L cells (GLP1), I cells (Cck), Enterochromaffin (EC) cells (Serotonin), X cells (Ghrelin), S cells (Secretin), K cells (GIP), delta cells (Somatostatin), and N cells (Neurotensin). Recent observations also from the study of enteroids indicate, that the previous characterization of cells making specific hormone-like precursors failed to realize that many of the EE subtypes could produce multiple hormones, some of which change by the state of differentiation^26–29^. It was established that enterochromaffin cells made both serotonin and tachykinin, with the latter present mostly in the crypt and serotonin expressed along most of the villus-crypt axis^30,31^. The ability to make multiple hormones was particularly true for the K and L EE subtypes^32,33^. Our finding of increased EE cells and serotonin is supported by a previous study in STZ-diabetic rat^15^. In addition, a recent study using proteomic profiling reported ChgA as one of the multiple elevated circulating factors in serum from T1D patients^22^. Our results together with previous findings suggest a need for characterization of the elevated EE cell subtypes together with their products in T1D to understand the contribution of the EE cell subtypes in T1D pathophysiology. Duodenal enteroids used in this study were established from pediatric T1D patients, who did not have diarrhea. Further characterization of the hormones made by individual EE cells in the UD and DF state in T1D enteroids from patients with diarrhea compared to those without diarrhea will be necessary to fully understand the changes in EE cells in T1D and their contribution to T1D diarrhea.

It is important, however, to put these T1D EE cell studies into the perspective of what happens in patients with T1D. There are surprisingly few descriptions published related to the EE cell population in the human intestine in T1D. Most detailed was an immunohistochemical comparison of 12 patients with long-standing T1D most with peripheral neuropathy and retinopathy with no statement if they had diarrhea, compared to between 28 and 40 HS based on the intestinal segments compared via endoscopic sampling of stomach, duodenum, and rectum^34^. In the stomach, enterochromaffin cells expressing serotonin were increased, while there was no significant change in the number of somatostatin and CCK/gastrin (antibody recognizes both) producing cells. In the duodenum, there were increased cells expressing serotonin, secretin, and CCK/gastrin, while there was no change in the number of somatostatin and GIP-producing EE cells, with secretin only present in villus and GIP only in the crypts. In the rectum, the number of serotonin and PYY-expressing cells was increased while there was no change in the somatostatin and pancreatic polypeptide-producing cells^34^. Thus our studies validate the increase in serotonin-producing EE cells in T1D. Serotonin via receptors 3 and 4 is known to induce intestinal fluid secretion and acts by elevating intracellular Ca^2+^, and ATP. Both Ca^2+^ and ATP are known to cause increased fluid secretion in T1D. We therefore suggest that it would be worth considering a trial with serotonin inhibitors to treat diarrhea in T1D patients^35–39^. Clinical trials to test this hypothesis are made even clearer since serotonin stimulates intestinal motility and long-term serotonin elevation alters transcriptional regulation as well^40^. Our studies did not examine in detail the mechanism of the increase in the EE. We expected that changes in neurogenin3 would have been present as it is the major transcription factor involved in EE cell development^41,42^. However, we were unable to find changes in *Ngn3* in T1D enteroids. A previous study using a time-resolved map of transcriptional changes predicted multiple other novel molecular regulators (Sox4, Rfx6, Tox3, Myt1, Runx1t1, and Zcchc12) of specific EE cell phenotypes. Our study using conventional differential gene expression analysis identified global changes in EE cell population. However, it does not distinguish specific gene expression in individual EE subspecies. Thus, detailed single-cell studies at different time intervals are needed to understand the mechanism of enrichment in EE cells in T1D enteroids.

The insights related to the pathophysiology of diarrhea that came from the comparison of T1D and HS enteroids in terms of protein expression support the previously demonstrated reduced NaCl absorption and NHE3 protein expression in differentiated T1D enteroids. The status of Cl^−^ secretion remains to be evaluated in T1D. The CFTR mRNA was reduced but only in the DF enteroids. Unlike the lack of changes in mRNA expression, SGLT1 protein was increased in both UD and DF T1D enteroids, suggesting a possible involvement of post-translational modification in *SGLT-1* in T1D. An increase in SGLT-1 protein expression was previously demonstrated in enteroids from obese patients with type 2 diabetes^43^.

While T1D intestine is associated with increased permeability and vice versa, no clear association with changes in tight junctional proteins has been described^17,44^. T1D enteroids did exhibit increased sensitivity to Ca^2+^ deprivation with increased permeability to 4 kDa dextran after EGTA exposure but not under baseline conditions. This is consistent with changes in the signaling pathways related to pore formation being affected in T1D. In addition, there are changes in tight junctional proteins, which were documented by bulk RNA sequencing which demonstrated changes in claudins and *ZO-2* and *ZO-3*, but not the adherens junction protein *E cadherin* in differentiated enteroids. In differentiated T1D enteroids the significant changes were : increased: *claudins 4,* and *12* and *ZO2* and *ZO3*; decreased: *claudin 23*. Claudins 4 and 23 are thought to contribute to the tightness of the paracellular pathway^45,46^. Of note, related to interpreting these changes is that claudins are expressed in much lower amounts in adults than in the pediatric intestine, the basis for the enteroids studied here. Claudin 23 has been shown to form complexes with Claudins 3 and 4 to increase the TJ pore thickness and reduce the tight junction pore diameter^46^. This observation was one of the first to indicate that claudins hetero-polymerize and function in groups as units to regulate tight junction function. Since *claudin 23* and *4* are altered in the opposite direction in T1D enteroids (reduced *claudin 23* and increased *claudin 4*), the altered permeability of T1D enteroids is potentially contributed to by the failure to form the claudin 23-claudin 3–4 complex. Increased *claudin 4* could also be explained by increased EE cells as they have been reported as a surface marker for EE cells in the mouse small intestine^47^. Left as important gaps are quantitating the protein expression of the claudins in T1D and understanding how these changes in claudins expression contribute to the intestinal pathophysiology of T1D.

The enteroid model of T1D established in this study has the potential to give information about multiple additional aspects of T1D enteric pathophysiology. We acknowledge that our findings are based on a small sample size. Nonetheless, even with a small sample size, T1D enteroids showed differences with the HS enteroids. Expanding the sample size of T1D enteroid lines used for comparison together with studies of proteomics and spatial transcriptomic approaches need to be applied as the next step in understanding the pathogenesis of GI aspects of T1D. In addition, the T1D enteroid model should be used to explore multiple poorly defined complications of T1D that are potentially related to the GI tract. One such example is a lack of understanding of intestinal Ca^2+^ absorption in human T1D, despite the frequency of osteopenia in T1D (~ 20% of T1D age 7–20 have osteopenia) and the evidence of reduced intestinal Ca^2+^ absorption in rodent models of T1D^48,49^. In addition, the approach described here will lead to obtaining T1D enteroids to address mechanisms to explain gender and ethnic differences in multiple clinical aspects of T1D that have only been identified epidemiologically until now. In addition, extending these approaches to the many other organs affected by T1D has the potential to provide similar insights into many additional aspects of T1D currently only described but not mechanistically understood.

## Methods

Chemicals and reagents were purchased from Thermo Fisher (Waltham, MA) or Sigma-Aldrich (St. Louis, MO) unless otherwise specified.

### Small intestine enteroid culture and monolayer formation

Duodenal biopsies were obtained from two T1D pediatric patients and three pediatric HS (Table 1) undergoing endoscopy following approval by the Johns Hopkins University Institutional Review Board (IRB00044373). Informed consent was provided by the parents or legal guardians. All procedures and methods were performed under approved guidelines and regulations. Duodenal biopsies were collected from HS screened with endoscopy for gastrointestinal symptoms and who had histologically normal duodenum. Enteroids were established via the Hopkins Conte Basic and Translational Digestive Diseases Research Core Center (NIH/NIDDK P30) following an established protocol^11,12,50^. Briefly, human enteroids were maintained as cysts embedded in Matrigel (Corning #356231; Corning, NY) in 24-well plates and cultured in Wnt3A, Rspon-1, and Noggin containing growth medium. For differentiation of enteroids, growth medium was replaced with Wnt3A and Rspon free medium for 5–6 days^11,12^. Enteroid monolayers were generated by plating fragmented enteroids (in 100 µl growth medium) onto 0.4 μm pore polyester (PET) membrane 24-well cell culture inserts (Transwell; Corning, USA or Millipore, USA) pre-coated with human collagen IV (30 μg/ml; Sigma-Aldrich, USA) following our established protocols^11,12^. The formation of an enteroid monolayer and changes with differentiation were monitored by the measurement of TEER using an epithelial volt/ohm meter (EVOM; World Precision Instruments)^12,51^.

**Table 1:** Clinical descriptions and origins of biopsies of pediatric healthy subjects (HS) and patients with T1D.

Duodenal lines	Known ailment	Gender	Ethnicity	Origin of biopsies
Patient 1	T1D	Female	White	Duodenum
Patient 2	T1D	Male	White	Duodenum
Healthy subject 1	Normal	Female	African American	Duodenum
Healthy subject 2	Normal	Male	White	Duodenum
Healthy subject 3	Normal	Male	White	Duodenum

### Brightfield imaging

Enteroids plated in Matrigel in 24 well plates were imaged on a Zeiss Axio Observer A1 inverted microscope (Zeiss, Oberkochen, Germany) with images captured on CellSense imaging software (Olympus, Tokyo, Japan). Images were viewed and processed using OlyVia (Olympus).

### RNA isolation, reverse transcription, and real-time PCR

Enteroids were harvested from Matrigel using a Cultrex harvesting solution or from monolayers on Transwells^11,51^. Total RNA from harvested enteroids was extracted using the PureLink RNA Mini Kit (Life Technologies, Carlsbad, CA) according to the manufacturer’s protocol. Complementary DNA was synthesized from 1 to 2 μg of RNA using SuperScript VILO Master Mix (Life Technologies). Quantitative real-time PCR was performed using Power SYBR Green Master Mix (Life Technologies) on a QuantStudio 12 K Flex real-time PCR system (Applied Biosystems, Foster City, CA). Each sample was studied in triplicate, and 5 ng RNA-equivalent complementary DNA was used for each reaction. The relative fold changes in mRNA levels of genes were determined by using the 2^-ΔΔCT^ method, with human 18S ribosomal RNA simultaneously studied and used as the internal control for normalization and shown as the fold change compared with relevant control. Commercially available primer pairs from OriGene Technologies (Rockville, MD) were used (Table 2).

**Table 2:** Gene-specific primers used for RT-PCR studies.

Gene	Forward	Reverse
Lgr5	CCTGCTTGACTTTGAGGAAGACCC	CCAGCCATCAAGCAGGTGTTCA
Ki67	GAAAGAGTGGCAACCTGCCTTC	GCACCAAGTTTTACTACATCTGCC
ALP	GCTGTAAGGACATCGCCTACCA	CCTGGCTTTCTCGTCACTCTCA
SI	ACATTCCAGCCAACTCCTGCTC	GCCCAATAAGCTGGCATGACTG
ATOH1	CCTTCCAGCAAACAGGTGAATGG	GAACGACGGGATAACATTGCGC
MUC2	ACTCTCCACACCCAGCATCATC	GTGTCTCCGTATGTGCCGTTGT
NGN3	CCTAAGAGCGAGTTGGCACTGA	AGTGCCGAGTTGAGGTTGTGCA
CHGA	GGTTCTTGAGAACCAGAGCAGC	GCTTCACCACTTTTCTCTGCCTC
LYZ	ACTACAATGCTGGAGACAGAAGC	GCACAAGCTACAGCATCAGCGA
AVIL	TGCGAACTCAGGCAGAGCACTA	CAGTGTTCTGGCTGCCATCACA
POU2F3	GCTGGAGAAGTTTGCCAAGACC	GTGAGATGGTGGTCTGGCTGAA
Lmx1a	CATCGAGCAGAGTGTCTACAGC	TGTCGTCGCTATCCAGGTCATG
Trachykinin (TAC1)	TTACTGGTCCGACTGGTACGAC	CAAAGAACTGCTGAGGCTTGGG
Glucagon (GLP1)	CGTTCCCTTCAAGACACAGAGG	ACGCCTGGAGTCCAGTTACTTG
Neurotensin	CAGCAGGGCTTTTCAACACTGG	CTCATACAGCTGCCGTTTCAGAA
Somatostatin (SST)	CCAGACTCCGTCAGTTTCTGCA	TTCCAGGGCATCATTCTCCGTC
Cholecystokinin (cck)	TGAGGGTATCGCAGAGAACGGA	CGGTCACTTATCCTGTGGCTGG
PYY	CGGACACGCTTCTTTCCAAAACG	TGGTTGGCAGATCTCCCAGGAG
Ghrelin	GCAGAGGATGAACTGGAAGTCC	CTCTTCCCAGAGGATGTCCTGA

### Bulk RNA sequencing library preparation and sequencing

Total RNA extracted from undifferentiated and 6-day-differentiated 3D enteroids was used for RNAseq. The concentration and integrity of the extracted total RNA were estimated using the Quant-iT™ RiboGreen® RNA Assay Kit (Thermo Fisher Scientific) and the Tapestation (Agilent) using the RNA High Sense chip, respectively. Approximately 500 ng of total RNA was used for downstream RNA-seq Library preparation. Library preparation and sequencing were performed at Discovery Life Sciences, Huntsville, AL. Briefly, Polyadenylated RNAs were captured using NEBNext Magnetic Oligo d(T)25 Beads. The NEBNext mRNA Library Prep Reagent Set for Illumina (New England BioLabs Inc., Ipswich, MA, USA) was then used to prepare individually bar-coded libraries as per the manufacturer's recommended protocol. Library concentration was assessed using the Quant-iT™ PicoGreen® dsDNA Reagent (Thermo Fisher Scientific), and the library quality was estimated by utilizing the Caliper LabChip GX DNA High Sense kit (PerkinElmer). Accurate quantification for sequencing applications was determined using the qPCR-based KAPA Biosystems Library Quantification Kit (Kapa Biosystems, Inc., Woburn, MA, USA). Paired-end sequencing (25 million, 100-bp, paired-end reads) was performed on Illumina NovaSeq 6000 sequencers (Illumina, Inc., San Diego, CA, USA) as per the manufacturer's recommended protocol.

### RNA-seq analysis

Raw sequence reads were checked for quality using the FASTQC package ver. 0.11.9; Illumina adapters and low-quality basepairs were trimmed using TrimGalore ver. 0.6.5 with default settings. Trimmed reads were aligned to human genome build GRCh38.98 using Gencode v40 and a count matrix was generated from the aligned reads using feature Counts. For alignment and counts, the Dragen Bio-IT Platform version 3.10 using specific RNA flags including duplicate marking was employed. Following count generation, we employed R version 4.1.0 and edgeR for normalization, testing procedures, PCA clustering, and differential expression analysis.

### Principal component analysis

PCA plots were done with ggplot2 R package using the normalized TPM counts for the undifferentiated and differentiated clusters.

### Heatmap DEG Volcano plot

Using the normalized counts from the DESeq2 analysis as the inputs, we created heatmaps for the top 50 differentially expressed genes (by p-adj values) using the pheatmap R package (R package version 1.0.12) and volcano plot depictions using the ggplot R package depicting the log2Foldchange on the x-axis and − log10(padj) on the y axis respectively.

### GSEA on pathways enrichment

Pathway enrichment analysis was done using over-representation analysis (ORA) and the gene set enrichment analysis (GSEA) ^52,53^. The input data for these analyses were the output normalized counts from DESeq2. The Gene Ontology pathway compendium gene set compiled by the MsigDB database was used for the GSEA analysis^54^. The GSEA analysis was performed on rank files comprised of gene symbols and the corresponding log2 fold changes for all the expressed genes; enrichment was considered significant for adjusted p-value FDR < 0.25. Functional analyses on the differentially expressed genes were further performed using Over Representation Analysis (ORA) to determine whether known biological functions or processes are overrepresented. We used the hypergeometric distribution as implemented by MSigDB, with significance achieved for adjusted p-value FDR < 0.05.

### Immunofluorescence staining and confocal imaging

Enteroid monolayers on Transwell inserts were washed with PBS and fixed in 4% paraformaldehyde for 30–45 min, incubated with 5% bovine serum albumin/0.1% saponin in phosphate-buffered saline for 1 h, and incubated with primary antibodies overnight at 4 °C. Primary antibodies used included: CHGA (rabbit polyclonal; 1:1000 dilution; Immunostar), DRA (SC-376187; mouse monoclonal, 1:500, Santa Cruz, Dallas, TX), NHE3 (NBP1-82,574; rabbit polyclonal; 1:1000, Novus, Littleton, CO), E-cadherin (Clone 36, rabbit polyclonal; 1:100, BD Biosciences) or SGLT-1 (rabbit polyclonal:1:250, Thermo Fisher Scientific). The next day, membranes were washed with PBS three times for 5 min each, and cells were then exposed to Alexa Fluor 488 goat anti-mouse and Alexa Fluor 594 goat anti-rabbit secondary antibodies (1:1000 dilution; Invitrogen, Waltham, MA, USA) or phalloidin (568 or 633) (Life Technologies) for 1 h. Nuclei were detected by incubating in DAPI for 15 min at room temperature. The membranes were washed with PBS three times for 5 min each and mounted on glass slides using ProLong Gold antifade mounting medium (Invitrogen, Waltham, MA, USA). Images were collected using × 40 oil (NA 1.25) immersion objective on an FV3000 confocal microscope (Olympus, Tokyo, Japan) with Olympus FV31S-SW and Fiji (ImageJ-2020) (NIH). For quantitative analysis, the same settings were used to image all samples.

### Immunoblotting

Enteroids were rinsed 3 times and harvested in phosphate-buffered saline by scraping. Cell pellets were collected by centrifugation, solubilized in lysis buffer (60 mmol/L HEPES, 150 mmol/L NaCl, 3 mmol/L KCl, 5 mmol/L EDTA trisodium, 3 mmol/L ethylene glycol-bis(β-aminoethyl ether)—*N*,*N*,*N′*,*N′*—tetraacetic acid, 1 mmol/L Na _3_ PO _4_, and 1% Triton X-100, pH 7.4) containing a protease inhibitor cocktail, and homogenized by sonication. Protein concentration was measured using the bicinchoninic acid method. Proteins were incubated with sodium dodecyl sulfate buffer (5 mmol/L Tris–HCl, 1% sodium dodecyl sulfate, 10% glycerol, 1% 2-mercaptoethanol, pH 6.8) at 37 °C for 10 min, 50 µg total protein was loaded per well and separated by sodium dodecyl sulfate–polyacrylamide gel electrophoresis on a 10% acrylamide gel, and transferred onto a nitrocellulose membrane. The blot was blocked with 5% nonfat milk, and probed with primary antibodies against DRA (SC-376187, 1:250), NHE3 (NBP1-82,574, 1:1000) or SGLT-1 (ab14686, rabbit, 1:1000, Abcam Waltham, MA), glyceraldehyde-3-phosphate dehydrogenase (G8795; mouse monoclonal, 1:5000, G8795; Sigma-Aldrich), overnight at 4 °C, followed by secondary antibody against mouse IgG (1:10,000) for 1 h at room temperature. All the primary antibodies used have been previously validated^55–57^. Protein bands were visualized and quantitated using an Odyssey system and Image Studio Lite Ver 4.0 (LI-COR Biosciences, Lincoln, NE, USA).

### FITC-dextran permeability assay

Intestinal barrier integrity of 2D duodenal enteroid monolayers was evaluated using fluorescein isothio-cyanate (FITC)-dextran (4 kDa) flux assays. Human enteroids monolayers on Transwell inserts were maintained as an UD culture in Wnt3A, Rspon-1, and Noggin-containing growth medium and differentiated for six days by removing Wnt3A and Rspon from the medium. To measure changes in permeability in DF enteroids, the monolayers were treated with 5 mM EGTA on the apical side of the Transwells and incubated in a 37 °C incubator for 2 h. After 2 h, EGTA was removed and 1:100 dilution of 4 mg/ml of 4 kDa FITC-dextran prepared in DF media was added to the apical side of the Transwell and incubated in 5% CO_2_/95% air atmosphere at 37 °C incubator for 2 h. After 2 h, media was collected from the apical and basal sides and assayed using a fluorescent plate reader at an excitation/emission wavelength of 490/520 nm. The TEER was measured before EGTA treatment and at the end of the experiment. No EGTA-treated monolayers were used as the controls. Concentrations of FITC-dextran in the collected media were calculated using a standard curve of FITC-dextran with known concentrations prepared in DF media.

### Organoid swelling assay

Fluid secretion was determined using the agonist-stimulated swelling assay in duodenal organoids^25,26^. Organoids were passaged weekly by mechanical disruption into single crypts that easily reseal and form new organoids. Approximately 50–200 organoids were plated in each well of a 24-well plate. On the day of the assay, the organoids were stained with calcein green, a fluorescent cell-permeable dye that is retained within living cells and facilitates live imaging by defining the plasma membrane. Then, forskolin (Fsk) 5 µM or ATP (5 µM) which causes an increase in intracellular adenylyl cyclase-cAMP or Ca^2+^ respectively were added to stimulate organoid swelling. Using live-cell microscopy, we observed a rapid expansion of both the lumen and total organoid surface area after the addition of the CFTR agonists^19^. DMSO-treated organoids showed no significant change in the surface area of organoids and hence were used as a control. Agonist-stimulated organoid swelling was calculated as described before using an organoid swelling analysis macro for ImageJ^26^. Briefly, to quantify swelling, total calcein-green surface areas were selected for each time point and expressed as a percentage increase of T = 0 min (set at 100%). The relative area increase was expressed per 10-min time interval, and measurements were generated for each condition (T = 0 to 60 min, baseline threshold set at 100%). The area under the curve (AUC) (T = 60 min, baseline 100%) was calculated using Prism.

### Statistical analysis

Differentially expressed genes (DEGs) between groups were assessed using the DESeq2 ver. 1.42.0 package in R^58^. Raw count data were used as input for DESeq2 analysis. The cutoff criteria for assessing the DEGs was |log2FC|> 1.5 and false discovery rate (FDR)-adjusted *p* value < 0.05.

Statistical analyses were performed using GraphPad Prism (version 8.01, GraphPad Software, San Diego, CA, USA). Data points represent means of individual experiments performed on different enteroid lines. Data presented are mean ± s.e.m. of at least three-five independent experiments, with an error bar equaling one s.e.m. Statistical significance was determined using the Student’s t-test or one-way ANOVA and post-analysis correction. Differences were considered statistically significant at *p* value ≤ 0.05.

All authors had access to the study data and reviewed and approved the final manuscript.

### Supplementary Information

Supplementary Information.

## Data Availability

The data and materials presented in this study are available on request from the corresponding author. The RNAseq normalized expression data will be available at GEO (www.ncbi.nlm.nih.gov/geo) under accession numbers GSE7759.
