# Supplementary material for: Type 1 diabetes human enteroid studies reveal major changes in the intestinal epithelial compartment
Source: Sci Rep. 2024 May 24;14:11911. doi: 10.1038/s41598-024-62282-x (PMC11126659; doi:10.1038/s41598-024-62282-x)

Type 1 Diabetes Human Enteroid Studies Reveal Major Changes In The Intestinal Epithelial Compartment

Vishwesh Bharadiya[1], Yan Rong[1], Zixin Zhang[1], Ruxian Lin[1], Anthony Lawrence Guerrerio[2], C. Ming Tse[1], Mark Donowitz[1,3], and Varsha Singh[1, #]

[1]Divisions of Gastroenterology & Hepatology, Department of Medicine,[2]Department of Pediatrics, [3]Department of Physiology, the Johns Hopkins University School of Medicine, Baltimore, Maryland, 21205, USA

[#]correspondance: Varsha Singh, Divisions of Gastroenterology & Hepatology, Department of Medicine, Johns Hopkins University School of Medicine, Baltimore, Maryland, 21205, USA

email: vsingh11@jhmi.edu

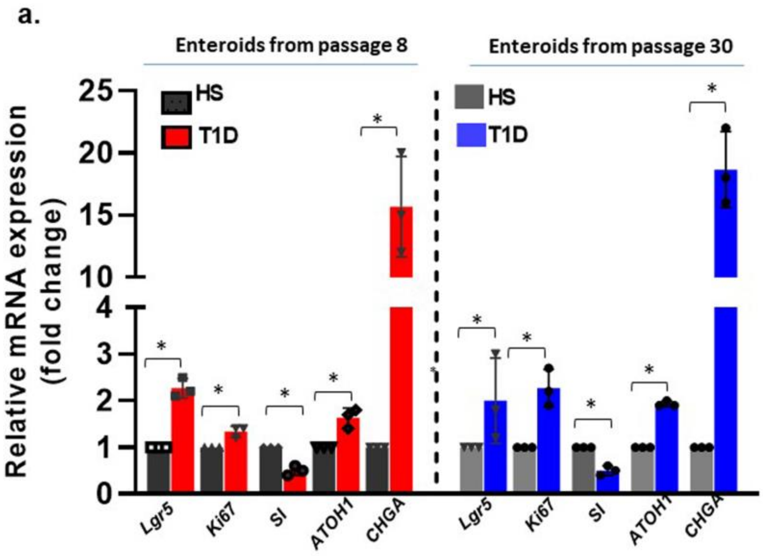

Fig S1. T1D enteroids retain pathological changes after multiple passaging

a. qRT-PCR analysis in duodenal enteroids from lower passage (p8) and higher passage (p30) showing differences between HS and T1D. T1D enteroids retain pathological differences in signature genes tested between the two groups. Results are shown as mean ± SEM. **P* <0.05 vs control. Analysis of differences was determined by Student's unpaired t-tests. N=3 independent experiment was performed on enteroids from 1 donor from each group.

Fig S2. The original images of blots.

Figure 3f

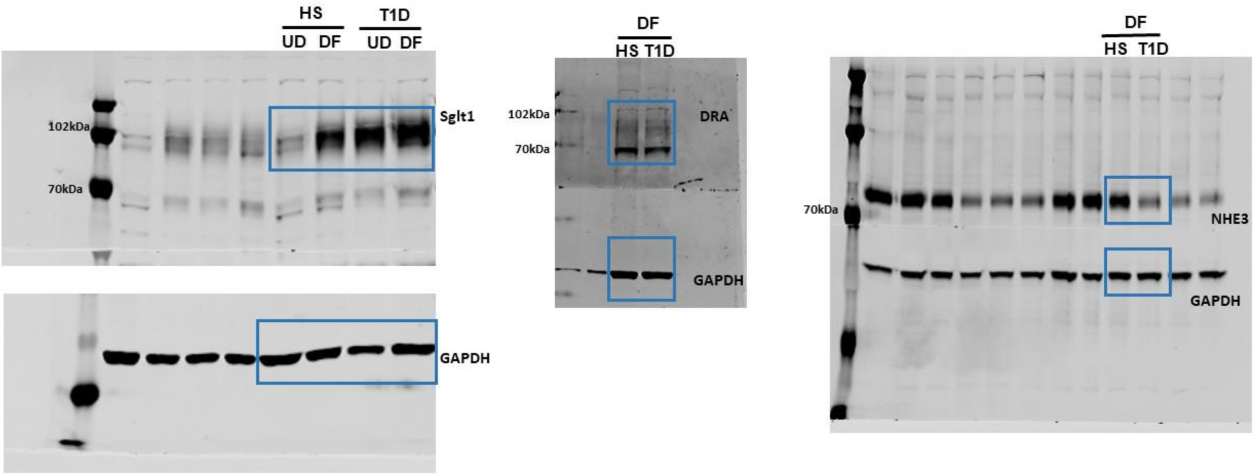

Supplement: Supplementary file 1 — Supplementary Information. [file 41598_2024_62282_MOESM1_ESM.pdf]
